# Concurrent presence of retinal hemorrhages in the setting of acute Vogt-Koyanagi-Harada syndrome - an unusual presentation

**DOI:** 10.1186/s12348-020-00203-5

**Published:** 2020-04-15

**Authors:** Sowkath Ali, Hnin Hnin Oo, Rupesh Agarwal, Peh Khaik Khee

**Affiliations:** 1 Department of Ophthalmology and Visual Sciences, Khoo Teck Puat Hospital, Yishun, Singapore; 2 Department of Ophthalmology, Tan Tock Seng Hospital, Novena, Singapore

To the Editor,

## Introduction

Vogt-Koyanagi-Harada syndrome (VKH), an autoimmune disorder, is an oculocutaneous meningeal syndrome commonly seen in pigmented race population characterized usually by bilateral granulomatous panuveitis, often associated with neurological, auditory, and cutaneous manifestations [1]. The incidence of VKH among Singaporean population in referral care centre was found to be 3% [2]. It usually presents in four phases—prodromal phase, acute uveitic phase, chronic or convalescent phase, and recurrent phase [3]. Exudative bilateral retinal detachments are usually noted in the acute uveitic phase which responds well to high dose of corticosteroids. The chronic or convalescent phase is characterized by the development of vitiligo, poliosis, and depigmentation of the choroid and usually occurs weeks after the acute uveitic phase [4]. The recurrent phase consists of a panuveitis with acute exacerbations of anterior uveitis [4]. We report a case of primary VKH syndrome in acute uveitic stage, with bilateral serous retinal detachment with multiple retinal hemorrhages which resolved completely with systemic corticosteroids. No specific cause for the presence of retinal hemorrhages was found, which also resolved with treatment. We present this case for its unusual presentation.

## Case report

**Fig. 1:**
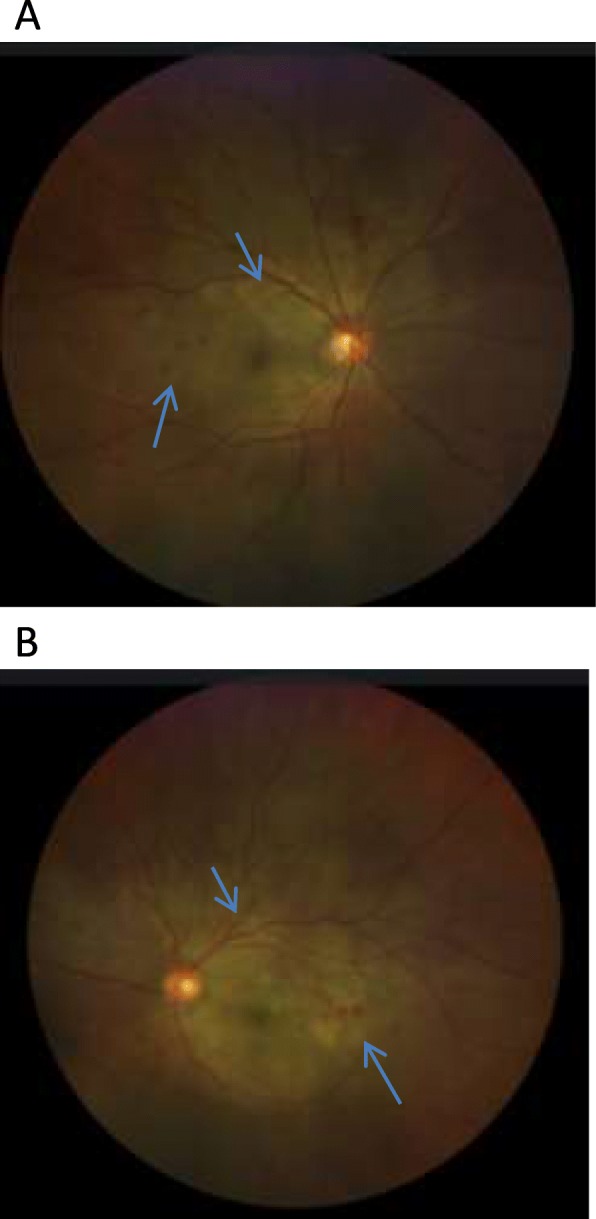
**a** Fundus photo of the right eye with arrowheads showing areas of subretinal fluid seen superotemporally, and areas of hemorrhages seen temporal to macula. **b** Fundus photo of the left eye showing areas of subretinal fluid superotemporally, and areas of hemorrhages infero temporal to macula

A 56-year-old lady was presented to the emergency clinic with acute onset of headache since 3 days followed by sudden progressive loss of vision in both eyes. No associated history of redness, photophobia, and floaters was noted. There was no evidence of any associated systemic illness. There was no uveitis-related systemic history in the form of joint pains, exposure to tuberculosis, exposure to pets, or fever with rashes. No previous history of any ocular surgery or trauma was noted. On examination, her best corrected visual acuity was counting fingers closely in both eyes; her intra ocular pressures were within normal limits. There was no relative afferent pupillary defect. Slit lamp examination showed anterior chamber reaction with cells occasionally in both eyes, no keratic precipitates were seen, lens was clear, and anterior vitreous face showed cells 1+ retrolentally. Fundus showed bilateral exudative retinal detachment with pockets of subretinal fluid in the posterior pole mainly superiorly and inferotemporally, with dot and blot hemorrhages seen around fovea, temporal to macula, and superior to disc. No evidence of vessel sheathing was seen (Fig. 1a, b).

**Fig. 2:**
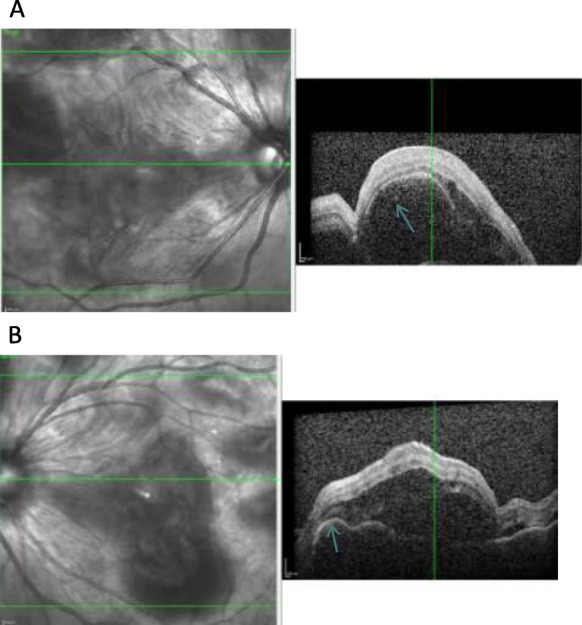
**a** SS-OCT Macula of the right eye showing area of serous detachment. **b** SS-OCT Macula of the left eye showing RPE undulations and subretinal fluid

**Fig. 3:**
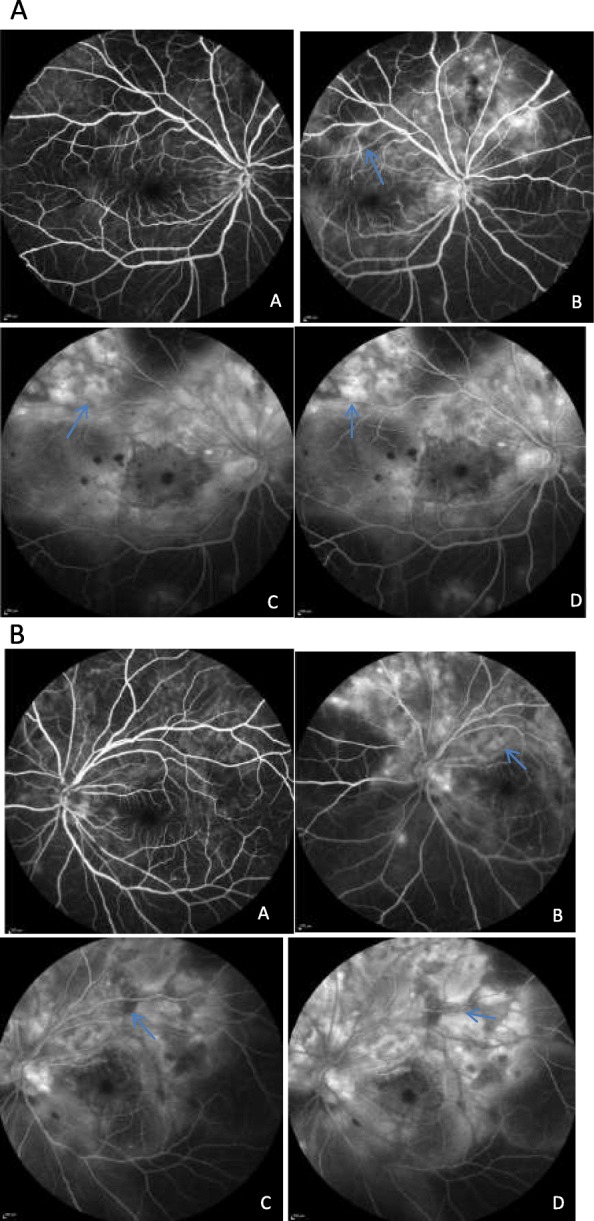
**a** Fluorescein angiography of the right eye at 1 min (**a**), 3.30 min (**b**), 11 min (**c**), and 15.30 min (**d**) showing early blocked fluorescence with pin-point leakage in the middle phase with late areas of placoid hyperfluoresence and with persistent areas of hypofluoresence corresponding to the hemorrhages. **b** Fluorescein angiography of the left eye at 1 min (**a**), 5 min (**b**), 10 min (**c**), and 16 min (**d**) showing early blocked fluorescence with pin-point leakage in the middle phase with late areas of placoid hyperfluoresence, and with persistent areas of hypofluoresence corresponding to the hemorrhages

Optical coherence tomography (OCT) macula showed areas of serous detachment (Fig. 2a) and RPE undulations with pockets of subretinal fluid (Fig. 2b). Fundus fluorescein angiography showed early blocked fluorescence with multiple pin-point leakages in the middle phases with placoid areas of hyperfluoresence in late phases (Fig. 3a, b). Clinical findings, OCT, and fluorescein angiography features are all typical of acute uveitic phase of VKH disease except for the presence of retinal hemorrhages. Differentials that were considered in our index case included leukemia, lymphoma, and syphilis. Magnetic resonance imaging (MRI) brain showed scattered foci of T2/FLAIR hyper intensities in the bilateral centrum semiovale, bilateral corona radiata, and subcortical and deep white matter of both frontal and parietal lobes. Full blood count was normal, and peripheral blood smear showed no significant morphologic abnormalities suggestive of hematopoietic tumor. Serological screen for syphilis, tuberculosis, and hepatitis viruses were negative.

**Fig. 4:**
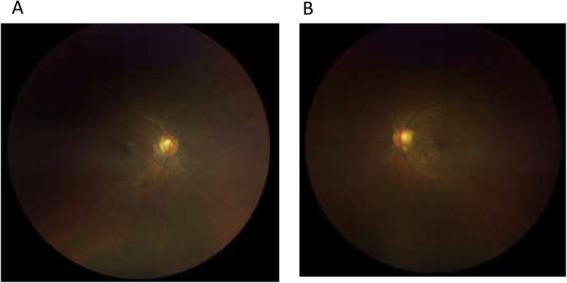
**a** Fundus photo of the right eye after treatment with high dose of corticosteroids with resolution of areas of subretinal fluid and also complete resolution of hemorrhages. **b** Fundus photo of the left eye after treatment with high dose of corticosteroids with resolution of areas of subretinal fluid and also complete resolution of hemorrhages

In view of the typical angiographic features of acute uveitic stage of VKH syndrome, systemic corticosteroids were started in the form of intravenous methylprednisolone 1 g/day for 3 days followed by oral prednisolone 1 mg/kg along with topical steroids. Patient responded well to the treatment with resolution of the exudative retinal detachments as well as the hemorrhages (Fig. 4). On subsequent follow-up, her best corrected visual acuity improved to 6/9 in both eyes. She was on close follow-up.

We present this case the presence of retinal hemorrhages in the setting of acute VKH that is quite unusual in the absence of associated systemic diseases.

## Discussion

The revised international classification of VKH is divided into incomplete, complete, and probable based on the systemic and ocular features [5]. Our index case did not have any skin, auditory, or neurological manifestations except for headache. MRI brain showed deep white matter lesions in the parietal lobes which were previously described as radiologic features of VKH syndrome [6]. She was found to have incomplete VKH syndrome based on the typical clinical and angiographic features in the absence of previous ocular surgery and trauma. However, presence of retinal hemorrhages was found to be unusual. Infective and hematological workup was within normal limits. There are case reports of atypical VKH syndrome noted in patients with leukemia [7, 8]. Choroidal infiltrations of leukemic cells with secondary RPE dysfunction can cause bilateral exudative retinal detachment mimicking VKH-like picture [9]. They can also present with auditory and neurological features as well as proliferative retinopathy with Roth spots, exudates, and hemorrhages. To our surprise, the peripheral smear was normal. Hence, we started the patient on high dose corticosteroids. Patient responded well with the resolution of both exudative retinal detachments and hemorrhages. We still cannot found out the cause of retinal hemorrhages and its resolution. Temporal association between retinal hemorrhages and VKH cannot be established in the index case; hence, the patient is in constant follow-up for any recurrences of same kind in the future. To our knowledge, retinal hemorrhages in acute uveitic phase of primary VKH syndrome are extremely rare. Retinal peripapillary hemorrhages along with optic disc edema have been previously reported in an observational case series of VKH syndrome in acute phase. Among 52 patients, 6 were found to have optic disc edema with hemorrhages secondary to anterior ischemic optic neuropathy [10]. Increase in retinal capillary fragility can occur in bullous rhegmatogenous detachment which can cause retinal hemorrhages, but our index case did show only few pockets of subretinal fluid which might not had increased the capillary fragility. Increase in venous pressure secondary to optic disc edema can also cause retinal hemorrhages, which was not found in our index case.

## Conclusion

We present this case due to its unusual concurrent presentation of hemorrhages and exudative retinal detachment in the acute uveitic stage of primary VKH syndrome with no specific cause for hemorrhages and its resolution with treatment. We would need to observe the patient closely for future recurrences with same features and subsequent systemic workup. No temporal causal association has been found between the retinal hemorrhages and posterior uveitis in our index case.

## Data Availability

Not applicable

## Abbreviations

VKH syndrome: Vogt-Koyanagi-Harada syndrome
RD: Retinal detachment
FFA: Fundus fluorescein angiogram
SS-OCT: Swept source optical coherence tomography
